# The Room Temperature Highly Sensitive Ammonia Gas Sensor Based on Polyaniline and Nitrogen-Doped Graphene Quantum Dot-Coated Hollow Indium Oxide Nanofiber Composite

**DOI:** 10.3390/polym13213676

**Published:** 2021-10-25

**Authors:** Sheng-Zhe Hong, Qing-Yi Huang, Tzong-Ming Wu

**Affiliations:** Department of Materials Science and Engineering, National Chung Hsing University, 250 Kuo Kuang Road, Taichung 402, Taiwan; gsn30628@gmail.com (S.-Z.H.); jordan20310687@gmail.com (Q.-Y.H.)

**Keywords:** polyaniline, nitrogen-doped graphene quantum dot, hollow indium trioxide nanofiber, composite, ammonia gas sensor

## Abstract

Hollow indium trioxide (In_2_O_3_) nanofibers fabricated via an effectively combined method of electrospinning and high-temperature calcination were coated with nitrogen-doped graphene quantum dots (N-GQDs) prepared by a hydrothermal process through electrostatic interaction. The N-GQD-coated hollow In_2_O_3_ nanofibers served as a core for the synthesis of polyaniline (PANI)/N-GQD/hollow In_2_O_3_ nanofiber ternary composites using in situ chemical oxidative polymerization. The chemical structure and morphology of the fabricated ternary composites were characterized using Fourier transform infrared, field-emission scanning electron microscopy, and transmission electron microscopy. The gas-sensing performances of the ternary composites were estimated by a homemade dynamic test system which was supplied with a real-time resistance acquisition platform at room temperature. The response value of the PANI/N-GQD/hollow In_2_O_3_ nanofiber sensor with a loading of 20 wt% N-GQD-coated hollow In_2_O_3_ nanofiber and an exposure of 1 ppm NH_3_ was 15.2, which was approximately more than 4.4 times higher than that of the PANI sensor. This ternary composite sensor was proved to be very sensitive in the detection of NH_3_ at a range of concentration between 0.6 ppm and 2.0 ppm at room temperature, which is crucial in the detection of hepatic or kidney disease in human breath. The PANI/N-GQD/hollow In_2_O_3_ nanofiber sensor also revealed higher selectivity and repeatability when exposed to 1.0 and 2.0 ppm NH_3_ at room temperature. Because of the excellent selectivity and repeatability in the detection of 1.0 and 2.0 ppm NH_3_ at room temperature achieved in this study, it is considered that the PANI/N-GQD/hollow In_2_O_3_ nanofiber composite sensor will be a favored gas-sensing material applied on human breath for the detection of hepatic or kidney disease.

## 1. Introduction

Human breath comprises various amounts of gas compounds in a range of concentration from a few ppt to thousands of ppm and a lot of humidity [1,2,3]. A composition change significantly depends on many issues, for example, age, gender, and health condition. A breakdown product of proteins, ammonia (NH_3_), normally converted into urea via the liver and evacuated by the kidneys, reveals specific relevance to hepatic or kidney disease [3,4,5,6,7], and when either of these two organs collapses, the concentration of NH_3_ increases from many hundred ppb when healthy up to several ppm. In accordance with a previous report, a concentration of NH_3_ less than 1.1 ppm is considered to be healthy, whereas greater than 1.6 ppm is regarded as unhealthy [8]. Since the concentrations of the borderline between healthy and unhealthy are unsure, an intermediate concentration range is identified for clarity. Hence, NH_3_ gas sensors are utilized to monitor the level of gas and sustain it within a specific limit.

Because electronic transfer between intrinsically conducting polymers (ICPs) and gas molecules rises as gas vapor adsorption increases, ICPs have been employed as an efficient material for sensing applications [9]. Among ICPs, polyaniline (PANI) is extensively employed for sensing applications due to its easy synthesis, exceptional doping/de-doping chemical reaction, high conductivity, outstanding environmental stability, and excellent responsivity to NH_3_ [10]. The incorporation of n-type metal oxide semiconductors, such as In_2_O_3_, SnO_2_, CeO_2_, CoFe_2_O_4_, or carbon-based materials into ICPs can improve the sensing performances of fabricated nanocomposites [11,12,13,14,15,16,17,18]. Xue et al. reported that a PANI/carbon nanotube (CNT) composite showed enhanced sensing performance and stability at room temperature as compared to that of pure PANI [12]. Yu et al. prepared a flexible PANI/SnO_2_ nanocomposite thin-film sensor based on PANI and SnO_2_. Their study revealed that the fabricated nanocomposites showed an improved response for ammonia and benzene gas with reduced recovery time [15]. Saleh et al. prepared an ammonia gas sensor by depositing a PANI/CeFe_2_O_4_ nanocomposite on flexible PET film. Their results showed excellent ammonia gas-sensing performance in the range of 1–50 ppm at room temperature [17]. Indium trioxide (In_2_O_3_), with high optical transparency and electrical conductivity, is widely used in liquid crystal displays and sensors [18]. Recently, Wu et al. used In_2_O_3_ to synthesize the PANI/graphene nanoribbon (GNR)/In_2_O_3_ composites with nanostructured conformation. Their investigations showed that the sensing performance at room temperature was extensively higher than that of pure GNR and In_2_O_3_ porous spheres [18]. The enhanced gas-sensing properties were attributed to an increase in the electron depletion layer by formation of a p–n junction between the p-type PANI and n-type In_2_O_3_.

Graphene quantum dots (GQDs), one of the zero-dimensional nanoscale carbon materials, contain a monolayer or few layers of carbon atoms in a closely packed honeycomb structure [19]. Since they are a group of graphene, GQDs show outstanding conductivity, exceptional biocompatibility, and low toxicity [20,21]. GQDs also display new occurrences attributable to quantum confinement and boundary effects and, thus, have lately received much attention [22,23,24,25]. GQDs are often used in cellular imaging, drug delivery, and photovoltaic and sensing devices. Consequently, several studies have focused on developing sensing systems for biological molecules. For example, Liu et al. [26] revealed that a fluorescent probe modified by GQDs can be used for the sensitive detection of ascorbic acid. Zhao et al. [27] investigated a GQD-based graphite electrode together with a single-stranded DNA probe for the sensitive and selective determination of many target molecules. Nevertheless, many developing topics with GQDs are critical in electrochemical sensors. According to previous investigations, doped carbon nanomaterials with heteroatoms could effectively alter their native properties, such as electronic individuality and natural features [28,29]. Nitrogen-doped GQDs (N-GQDs) have been reported to have outstanding electrocatalytic capability [30].

Besides the selection of functional materials, the dimension and structure of the sensor materials also perform a significant role in sensing performance. Consequently, numerous studies have been dedicated to this topic [31,32,33]. For example, Rosmalini et al. reported the preparation of filled and hollow well-aligned electrospun SnO_2_ nanofibers at 150 °C used as a H_2_ gas sensor [31]. Their results indicated that the granular hollow SnO_2_ nanofibers exhibited the highest response. Cheng et al. prepared SnO_2_ hollow nanofibers with porous structures fabricated using electrospinning and calcination procedures for outstanding gas sensor application [32]. However, as far as we know, no report on PANI, N-GQD, or hollow In_2_O_3_ nanofiber composites has been published.

This work describes the fabrication of a new ternary nanocomposite based on the conducting polymer PANI, hollow In_2_O_3_ nanofiber, and N-GQD as electrode materials used as gas sensors to detect ammonia in the concentration range of 1.0–1.6 ppm in human breath. Accordingly, the fabricated material is expected to display improved gas-sensing properties and excellent repeatability. The structure and gas-sensing properties of the synthesized composites are classified in the following discussion.

## 2. Materials and Methods

### 2.1. Materials

Aniline monomer, citric acid (CA, >98%), indium (III) nitrate hydrate (In(NO_3_)_3_, >99.9%), sulfuric acid (>98%), and urea (>98%) were obtained from Sigma-Aldrich Chemical Company (St. Louis, MO, USA). Ammonium persulfate (APS, >98%), isopropyl alcohol (>98%), and polyvinylpyrrolidone (PVP) were purchased from JT-Baker Chemical Company (Phillipsburg, NJ, USA). All chemicals were used without purification.

### 2.2. Preparation of Graphene Nanoribbon and Indium Trioxide

The N-GQDs, prepared by a hydrothermal process using urea as a nitrogen source, were synthesized according to previous reports [30,34]. First, 0.18 g urea and 0.21 g CA were mixed under stirring at room temperature for 30 min in a 10 mL beaker. Then, the mixture was transferred to a poly(tetrafluoroethylene) reactor and heated at 160 °C for 4 h. The reactants were further treated by adding ethanol to the solution and centrifuging at 5000 rpm for 2 h to obtain the N-GQD specimens. Finally, the product was washed with distilled water (DI-water) and subsequently purified by a dialysis bag for 24 h.

Indium nitrate hydrate was used as an indium source to prepare the hollow In_2_O_3_ nanofibers. In a typical fabrication process, 3.5 g PVP and 1.1 g In(NO_3_)_3_ were mixed in 12 mL ethyl alcohol and 10.6 mL DMF, and the solution was stirred to dissolve PVP and In(NO_3_)_3_ completely for 10 h. Then, the solution was filled into a 20 mL syringe including a 0.5 mm diameter metallic needle for electrospinning. A 20 kV high-voltage power was applied to the metallic needle tip, set at a feeding rate of 0.3 mL/h, and the distance between the needle and the collector was about 15 cm. After the 24 h electrospinning process, the obtained composite nanofibers were thermally annealed to fabricate hollow In_2_O_3_ nanofibers [29,32]. The thermal annealing process using calcination was operated at 800 °C for 3 h with a heating rate of 5 °C/min.

### 2.3. Synthesis of Polyaniline/Nitrogen-Doped Graphene Quantum Dot/Hollow Indium Trioxide Nanofiber Composites

**Scheme 1:**
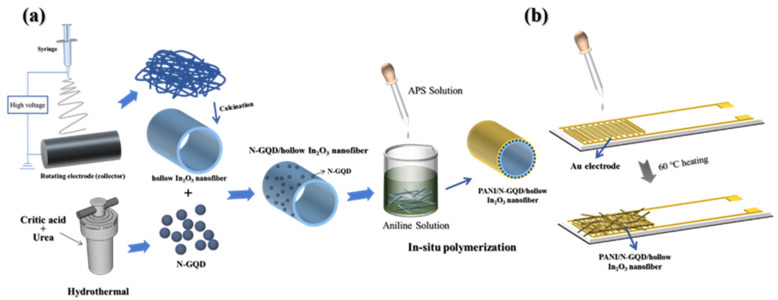
The schematic illustration of (**a**) preparation of hollow In_2_O_3_ nanofibers, N-GQDs, and PANI/N-GQD/hollow In_2_O_3_ nanofiber composites and (**b**) preparation of the PANI/N-GQD/hollow In_2_O_3_ nanofiber sensor.

The polyaniline (PANI)/N-GQD/hollow indium trioxide nanofiber composites were prepared using in situ chemical oxidative polymerization. Scheme 1a shows a schematic illustration of this preparation. In a typical preparation process, a certain weight ratio of N-GQD powders was dispersed in DI-water, mixed with hollow In_2_O_3_ nanofibers through electrostatic interaction, and sonicated for 1 h. The reactants were further treated by adding ethanol to the solution and centrifuging at 5000 rpm for 2 h to obtain the N-GQD-coated hollow In_2_O_3_ nanofiber specimens. Finally, the product was washed with DI-water and subsequently purified by a dialysis bag for 24 h. The obtained N-GQD/hollow In_2_O_3_ nanofiber was added to 50 mL HCl solution and sonicated for 2 h. After that, the aniline monomer was added to the dispersed solution of N-GQD/hollow In_2_O_3_ nanofiber and stirred for 1 h. Subsequently, ammonium persulfate dissolved in 20 mL HCl solution was progressively added to the mixed solution of aniline monomer/N-GQD/hollow In_2_O_3_ nanofiber and reacted for 3 h at 0 °C. The obtained PANI/N-GQD/hollow In_2_O_3_ nanofiber composite was filtered, rinsed several times with methanol and DI-water, and vacuum dried at 60 °C for 24 h. For comparison, the PANI/hollow In_2_O_3_ nanofiber composites were synthesized using the same method as for the PANI/N-GQD/hollow In_2_O_3_ nanofiber composites.

### 2.4. Analytical Procedures

The molecular structures of the fabricated PANI/N-GQD/hollow In_2_O_3_ nanofiber composites were determined by Fourier transform infrared (FTIR), Raman, and wide-angle X-ray diffraction (WAXD). FTIR spectra were determined with a PerkinElmer Spectrum One spectrometer (Waltham, Massachusetts, USA) in a range of 400–4000 cm^−1^. Raman spectra were obtained on a Renishaw system 1000 (Renishaw Inc., West Dundee, IL, USA) using an argon ion laser performing at 514.5 nm with a CCD detector. WAXD measurement experiments were operated using an X-ray diffractometer (Bruker D8, BRUKER AXS, Inc., Madison, WI, USA) equipped with a Ni-filtered Cu Kα radiation. The WAXD experiments were recorded in the range of 2θ = 1.5°–40° with an increment of 1°/min. The morphology of all samples was classified using field-emission scanning electron microscopy (FESEM) and transmission electron microscopy (TEM). The measurement of FESEM was carried out using a JEOL JSM-6700F field-emission instrument (JEOL Ltd., Tokyo, Japan). All specimens were coated with gold to avoid charging. The measurement of TEM was carried out using JEOL JEM-2010 (JEOL Ltd., Tokyo, Japan). Samples for TEM measurement were prepared by a Reichert Ultracut ultramicrotome. The particle size distribution was obtained using a Zetasizer Nano ZS instrument (Malvern Instruments Ltd., Worcestershire, UK). The specific surface area was determined using N_2_ sorption isotherms by the BET and BJH methods using a gas sorption analyzer (Quantachrome AutoSorb IQ, Montgomeryville, PA, USA).

Scheme 1b shows a schematic illustration of the sensor preparation. The gas-sensing properties of the sensors were evaluated by a homemade dynamic test system equipped with a real-time resistance acquisition platform at 25 °C and a relative humidity of 40 ± 5%. The gas concentrations of NH_3_ samples and interfering gas samples, including CH_3_OH, C_2_H_5_OH, and C_3_H_6_O, were investigated by altering the mixing ratio of nitrogen and test samples. The sensor response is given using the equation of R = R_g_/R_a_, where R_g_ and R_a_ are the sensor resistances in test gases and air, respectively [30]. The slope of the response–concentration fitting curve is designated as the sensitivity of the sensors. The response time and recovery time were calculated as the time taken for the sensor to attain 90% of total resistance change from its initial resistance.

## 3. Results

### Synthesis, Structure, and Morphology of PANI/N-GQD/Hollow In_2_O_3_ Nanofiber Composites

**Figure 1:**
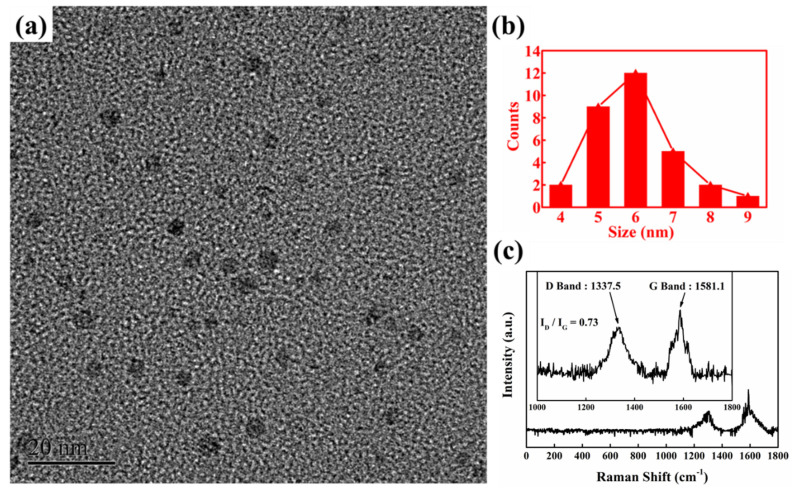
(**a**) TEM images of N-GQD, (**b**) particle size distribution of N-GQD, and (**c**) Raman spectrum of N-GQD.

A characteristic TEM micrograph of N-GQDs is shown in Figure 1a. The average diameter of the N-GQDs, determined using a Nano ZS instrument, was about 6 nm (Figure 1b). Raman spectra were used to obtain the absorption bands of the N-GQDs and are shown in Figure 1c. In this figure, two strong absorption bands at 1581 cm^−1^ (G mode) and at 1338 cm^−1^ (D mode) are observed [34].

**Figure 2:**
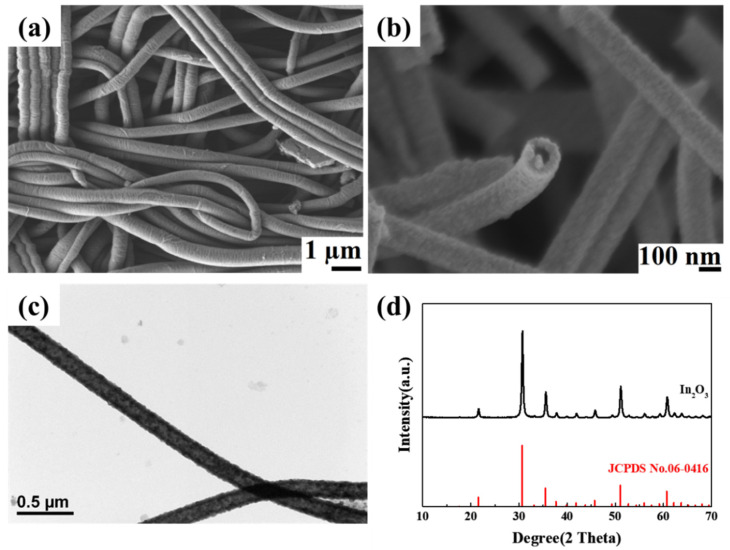
(**a**) SEM images of In_2_O_3_/PVP electrospun fibers; (**b**) SEM images of hollow In_2_O_3_ nanofibers after 800 °C calcination; (**c**) TEM images of hollow In_2_O_3_ nanofibers; and (**d**) WAXD pattern of hollow In_2_O_3_ nanofibers.

The morphology and structure of the fabricated In_2_O_3_ nanofibers were determined using WAXD, FESEM, and TEM. Figure 2a exhibits a typical FESEM micrograph of an electrospun In (NO_3_)_3_/PVP nanofiber, which displays a continuous fibrous morphology with smooth and uniform surfaces. The average diameter is about 540 nm. After calcination in air, the diameter of the In_2_O_3_ nanofibers decreased drastically to 165 nm, and the surfaces became coarse, as presented in Figure 2b. In addition, the microstructure of hollow In_2_O_3_ nanofibers was further identified by TEM, as illustrated in Figure 2c. The crystalline structure of the hollow In_2_O_3_ nanofibers was determined using a WAXD technique. The WAXD diffraction pattern of the hollow In_2_O_3_ nanofibers, as presented in Figure 2d, exhibits five strong diffraction peaks at 2*θ* = 21.7°, 30.6°, 35.4°, 51.2°, and 60.7°, assigned to the (211), (222), (400), (440), and (622) crystalline planes of In_2_O_3_, respectively. This finding suggests that the crystalline structure of the hollow In_2_O_3_ nanofibers is a cubic phase [18].

**Figure 3:**
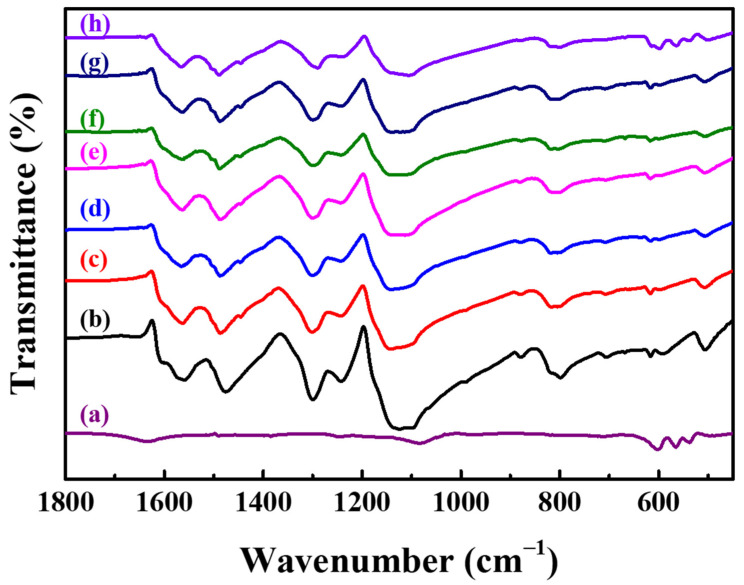
FTIR spectra of (**a**) hollow In_2_O_3_ nanofiber, (**b**) neat PANI polymer matrix, (**c**) 2.5 wt% PANI/hollow In_2_O_3_ nanofiber, (**d**) 5 wt% PANI/hollow In_2_O_3_ nanofiber, (**e**) 10 wt% PANI/hollow In_2_O_3_ nanofiber, (**f**) 15 wt% PANI/hollow In_2_O_3_ nanofiber, (**g**) 20 wt% PANI/hollow In_2_O_3_ nanofiber, and (**h**) 25 wt% PANI/hollow In_2_O_3_ nanofiber composites.

The chemical structure and morphology of the fabricated polyaniline (PANI)-coated hollow In_2_O_3_ nanofibers synthesized using in situ chemical oxidative polymerization were identified using the FTIR, FESEM, and TEM methods. Figure 3 shows the FTIR spectra of PANI-coated composites with various weight ratios of hollow In_2_O_3_ nanofiber. The FTIR data of neat PANI and hollow In_2_O_3_ nanofiber were also presented in this figure. The absorption peaks of hollow In_2_O_3_ nanofiber occurring at 538, 567, and 600 cm^−1^ were attributed to the In–O–In stretching vibration [35]. The characteristic peak of PANI observed at 800 cm^−1^ was attributed to the C–N^●+^ stretching vibration, and the absorption band at 1240 cm^−1^ was ascribed to a C–H out-of-plane bending vibration of the 1,4-disubstituted aromatic rings [36]. The absorption peaks at 1112 and 1294 cm^−1^ were assigned to the C=N and C–N stretching vibrations, respectively [36]. The FTIR spectra of PANI coated with significant amounts of hollow In_2_O_3_ nanofiber were approximately identical to those of pure PANI, indicating that the surface of the hollow In_2_O_3_ nanofibers was coated with PANI to form PANI/hollow In_2_O_3_ nanofiber composites. It was also found that the FTIR spectra of PANI/hollow In_2_O_3_ nanofiber composites with higher contents of hollow In_2_O_3_ nanofiber show absorption peaks occurring at 538, 567, and 600 cm^−1^, which were attributed to the In–O–In stretching vibration. This result reveals that the surface of partial hollow In_2_O_3_ nanofibers was not completely coated with PANI.

**Figure 4:**
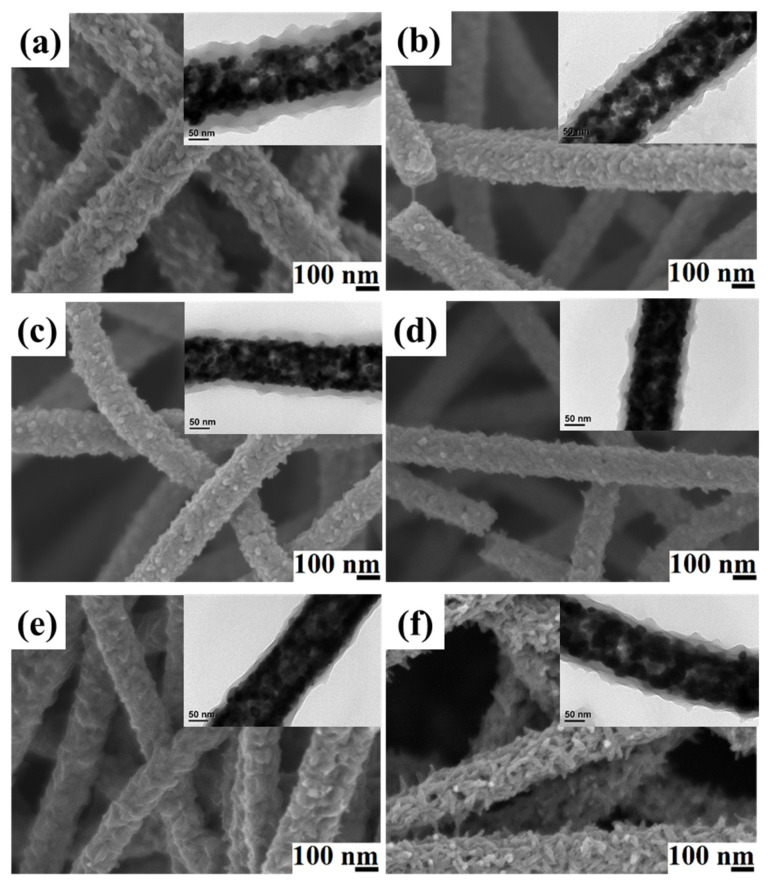
SEM and TEM images of (**a**) 2.5 wt% PANI/hollow In_2_O_3_ nanofiber, (**b**) 5 wt% PANI/hollow In_2_O_3_ nanofiber, (**c**) 10 wt% PANI/hollow In_2_O_3_ nanofiber, (**d**) 15 wt% PANI/hollow In_2_O_3_ nanofiber, (**e**) 20 wt% PANI/hollow In_2_O_3_ nanofiber, and (**f**) 25 wt% PANI/hollow In_2_O_3_ nanofiber composites.

**Figure 5:**
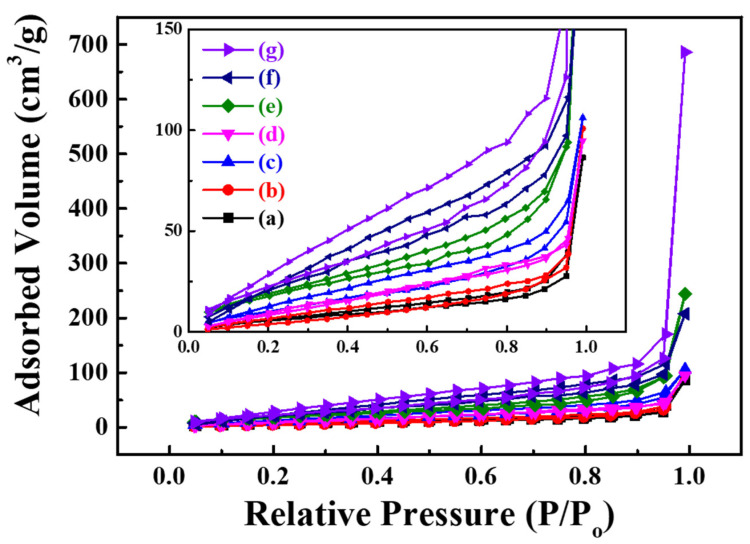
The BET curves of (**a**) neat PANI polymer matrix, (**b**) 2.5 wt% PANI/hollow In_2_O_3_ nanofiber, (**c**) 5 wt% PANI/hollow In_2_O_3_ nanofiber, (**d**) 10 wt% PANI/hollow In_2_O_3_ nanofiber, (**e**) 15 wt% PANI/hollow In_2_O_3_ nanofiber, (**f**) 20 wt% PANI/hollow In_2_O_3_ nanofiber, and (**g**) 25 wt% PANI/hollow In_2_O_3_ nanofiber composites.

Figure 4 shows the FESEM and TEM images of PANI/hollow In_2_O_3_ nanofiber composites with various loading ratios of hollow In_2_O_3_ nanofibers. By adding the hollow In_2_O_3_ nanofiber to PANI, the diameters of the PANI/hollow In_2_O_3_ nanofiber composites decreased as the weight ratio of hollow In_2_O_3_ nanofiber increased to 20 wt%. Notably, in Figure 4a, the diameters of the PANI/hollow In_2_O_3_ nanofiber composites decreased from 180–200 nm to 140–160 nm for 20 wt% hollow In_2_O_3_ nanofiber. The decreasing diameter in the PANI/hollow In_2_O_3_ nanofiber composites can be attributed to the increasing weight ratio of hollow In_2_O_3_ nanofiber, which reduces the ratio of aniline monomer/hollow In_2_O_3_ nanofiber and possibly gives more active sites for the absorption and polymerization of aniline monomers on the surface of the hollow In_2_O_3_ nanofiber, thereby decreasing the coating thickness of PANI layers. The data of the coating thickness of the PANI layer for the PANI/hollow In_2_O_3_ nanofiber composites, calculated using TEM images, is listed in Table 1. As the content of hollow In_2_O_3_ nanofiber increases, the diameter of PANI/hollow In_2_O_3_ nanofiber composites slightly increases, which can be ascribed to the aggregation effect. Figure 5 shows the specific surface area of the PANI/hollow In_2_O_3_ nanofiber composites with various loading ratios of hollow In_2_O_3_ nanofiber. The data of the specific surface area for the PANI/hollow In_2_O_3_ nanofiber composites are also recorded in Table 1. It is clear that the specific surface area increases with the increase in weight ratio of hollow In_2_O_3_ nanofiber to 20 wt%. This observation is in good agreement with the FESEM/TEM images.

**Figure 6:**
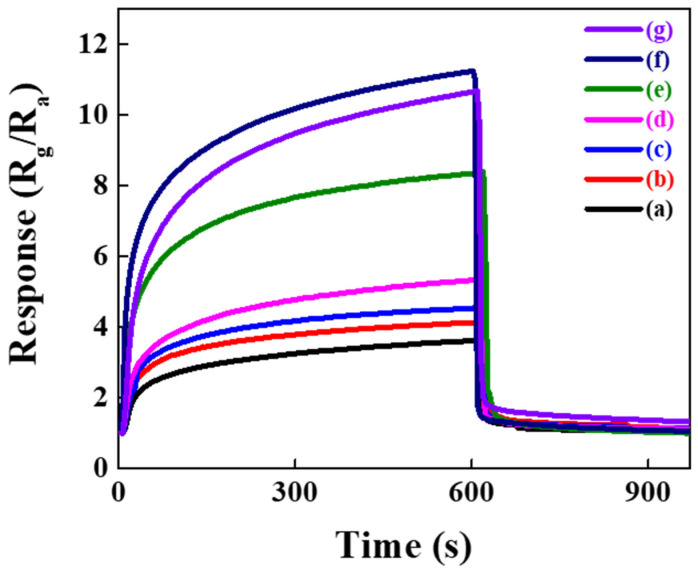
The response curves of (**a**) neat PANI polymer matrix, (**b**) 2.5 wt% PANI/hollow In_2_O_3_ nanofiber, (**c**) 5 wt% PANI/hollow In_2_O_3_ nanofiber, (**d**) 10 wt% PANI/hollow In_2_O_3_ nanofiber, (**e**) 15 wt% PANI/hollow In_2_O_3_ nanofiber, (**f**) 20 wt% PANI/hollow In_2_O_3_ nanofiber, and (**g**) 25 wt% PANI/hollow In_2_O_3_ nanofiber composites with exposure of 1 ppm NH_3_ at room temperature.

**Figure 7:**
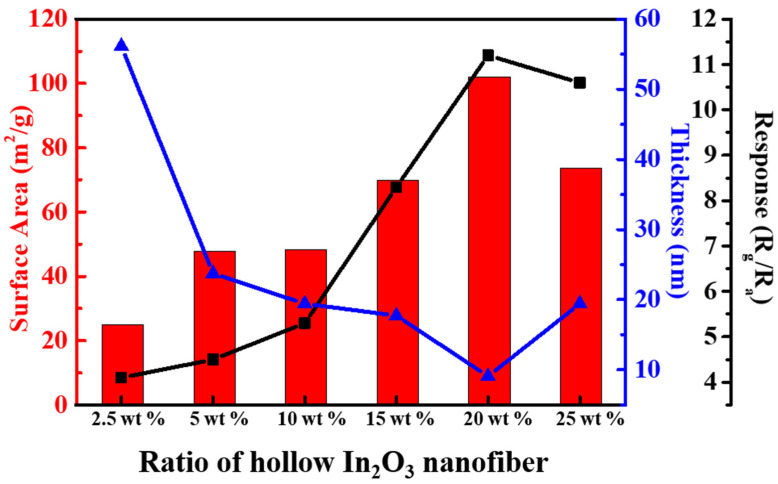
The surface area and thickness of PANI-coated hollow In_2_O_3_ nanofiber and the response versus the loading of hollow In_2_O_3_ nanofiber of PANI/hollow In_2_O_3_ nanofiber composites.

**Figure 8:**
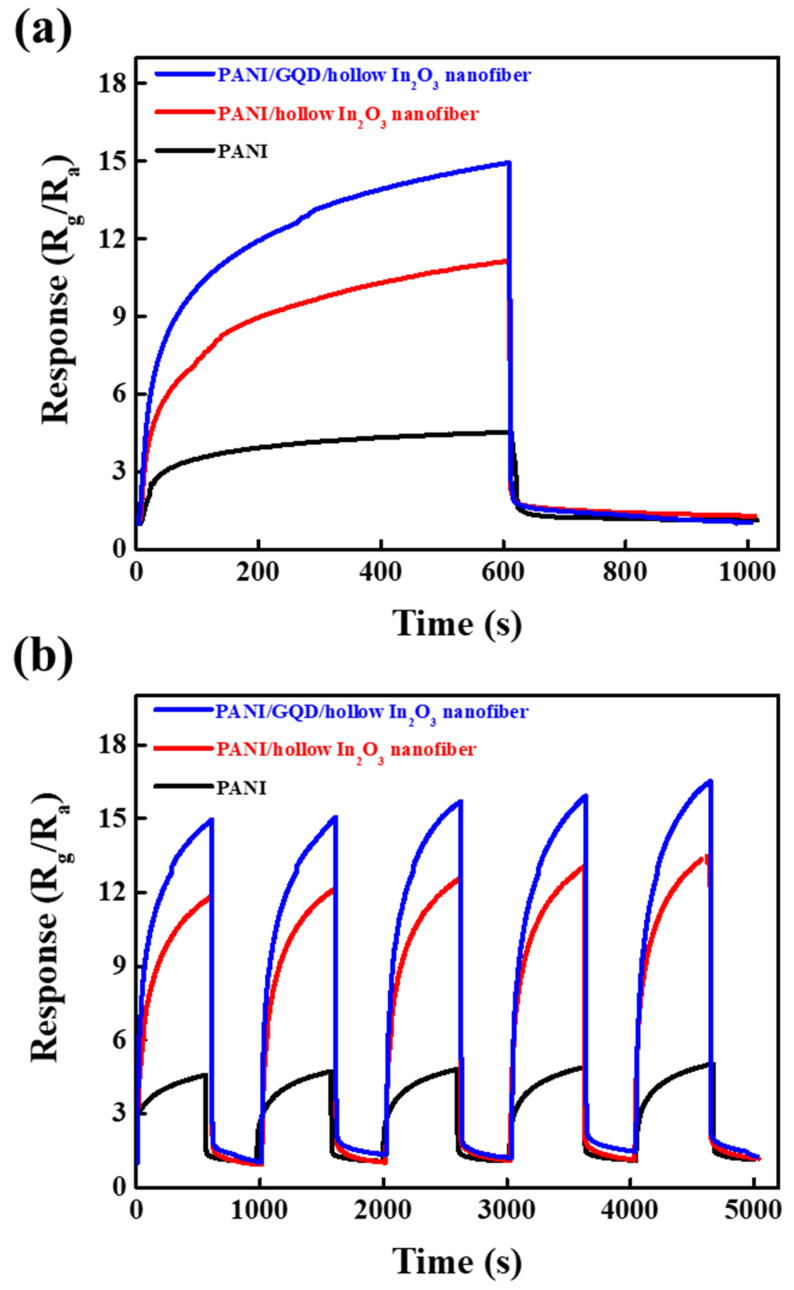
(**a**) The response curves and (**b**) the sensing repeatability and reversibility of neat the PANI polymer matrix, 20 wt% PANI/hollow In_2_O_3_ nanofiber, and 20 wt% PANI/GQD/hollow In_2_O_3_ nanofiber composites with exposure of 1 ppm NH_3_ at room temperature.

The response and recovery attributes of neat PANI and various hollow In_2_O_3_ nanofiber ratios of PANI/hollow In_2_O_3_ nanofiber sensors were comparatively investigated to estimate the effect of the weight ratio of hollow In_2_O_3_ nanofiber on the NH_3_-sensing performance of the PANI/hollow In_2_O_3_ nanofiber sensor. The dynamic response–recovery curves of the composite sensors, with exposure of 1 ppm NH_3_ at room temperature, are presented in Figure 6. It was observed that the PANI/hollow In_2_O_3_ nanofiber sensor reacted with an improvement in resistance when exposed to NH_3_ and the resistance dropped down to the original state after the NH_3_ was substituted with dry air. This result reveals a distinctive performance of the PANI/hollow In_2_O_3_ nanofiber composite film and an excellent reversibility of the PANI/hollow In_2_O_3_ nanofiber sensor. Particularly, the PANI/hollow In_2_O_3_ nanofiber sensor exhibited much higher response values than the pure PANI, indicating that the hollow In_2_O_3_ nanofiber plays a promising role in NH_3_-sensing measurements. It was also noticed that the PANI/hollow In_2_O_3_ nanofiber sensor with 20 wt% hollow In_2_O_3_ nanofiber loading achieved a maximum response which was far superior to other composite specimens. In order to estimate the effect of different weight ratios of hollow In_2_O_3_ nanofiber on the NH_3_-sensing performance of the PANI/hollow In_2_O_3_ nanofiber sensor, the plot of the specific surface area, the coating thickness of the PANI layer, and the response values for the PANI/hollow In_2_O_3_ nanofiber composites versus the adding ratios of hollow In_2_O_3_ nanofiber is shown in Figure 7. It is clear that the 20 wt% hollow In_2_O_3_ nanofiber loaded PANI/hollow In_2_O_3_ nanofiber composite possesses the highest specific surface area, the smallest coating thickness of the PANI layer, and the highest response values of the PANI/hollow In_2_O_3_ nanofiber composites. Therefore, 20 wt% hollow In_2_O_3_ nanofiber loading was used as the optimal loading ratio to fabricate the PANI/N-GQD/hollow In_2_O_3_ nanofiber composites. Figure 8a illustrates the response of PANI, 20 wt% PANI/hollow In_2_O_3_ nanofiber, and 20 wt% N-GQD-coated hollow In_2_O_3_ PANI/N-GQD/hollow In_2_O_3_ nanofiber. The response value of pure PANI was about 3.6, but with the loading of 20 wt% hollow In_2_O_3_ nanofiber into PANI the response value was extensively enhanced to 11.2. The response value of the PANI/N-GQD/hollow In_2_O_3_ nanofiber sensor was 15.2 with the loading of coated N-GQD on the surface of the hollow In_2_O_3_ nanofiber. The improvement of the sensing performance was ascribed to the existence of a p–n junction between the p-type PANI and n-type N-GQD-coated hollow In_2_O_3_ nanofiber [18,36]. The sensing reversibility and repeatability of the PANI/N-GQD/hollow In_2_O_3_ nanofiber sensor to 1.0 ppm NH_3_ are shown in Figure 8b. The response value of the PANI/N-GQD/hollow In_2_O_3_ nanofiber gas sensor came back to the initial response value after exposure to 1.0 ppm NH_3_. This characteristic behavior of response and recovery to 1.0 ppm NH_3_ confirmed excellent reproducibility in the process of five continues cycles. This result suggests that the PANI/N-GQD/hollow In_2_O_3_ nanofiber sensor has good repeatability.

**Figure 9:**
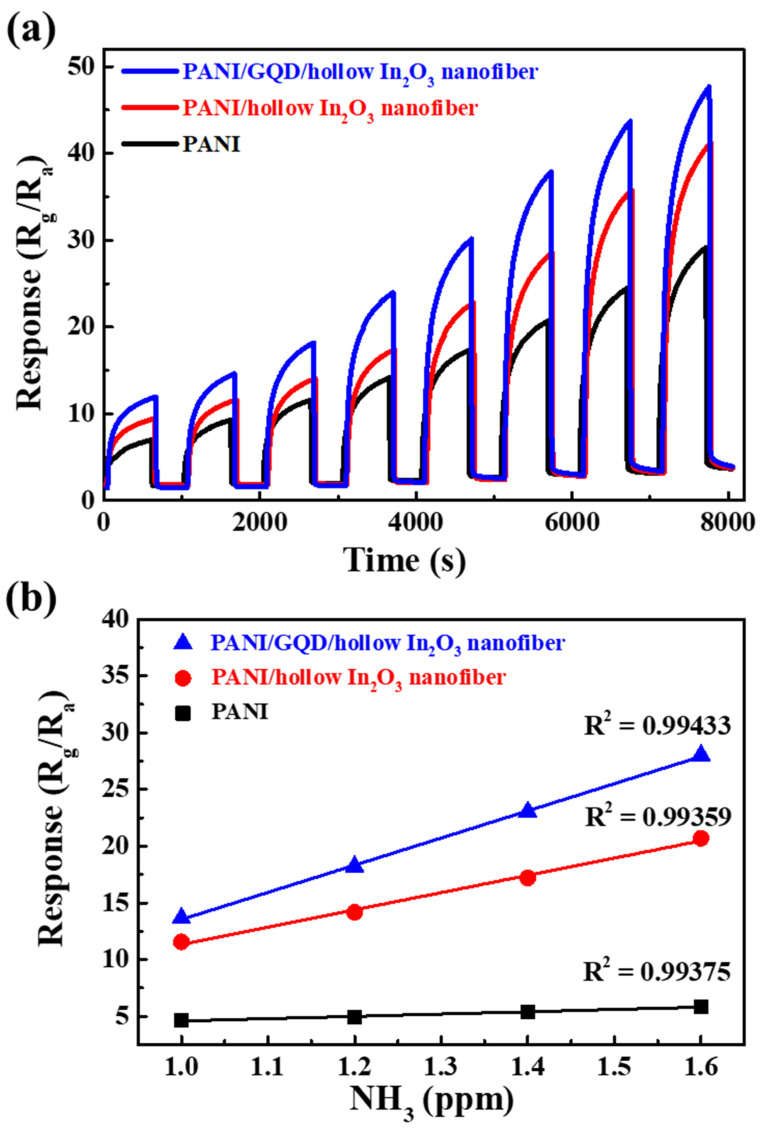
(**a**) Dynamic response–recovery curves and (**b**) response–concentration fitting curves of the PANI/GQD/hollow In_2_O_3_ nanofiber sensor.

**Figure 10:**
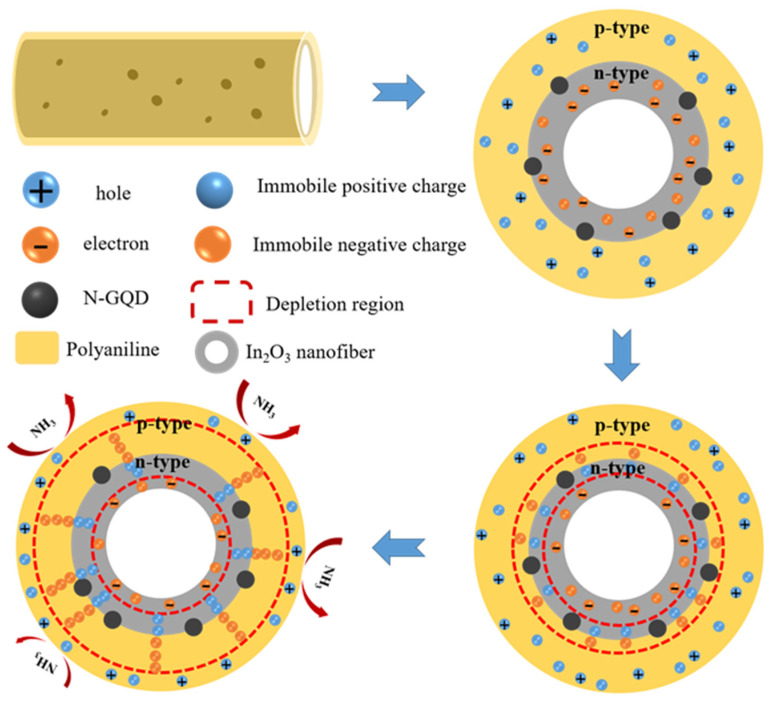

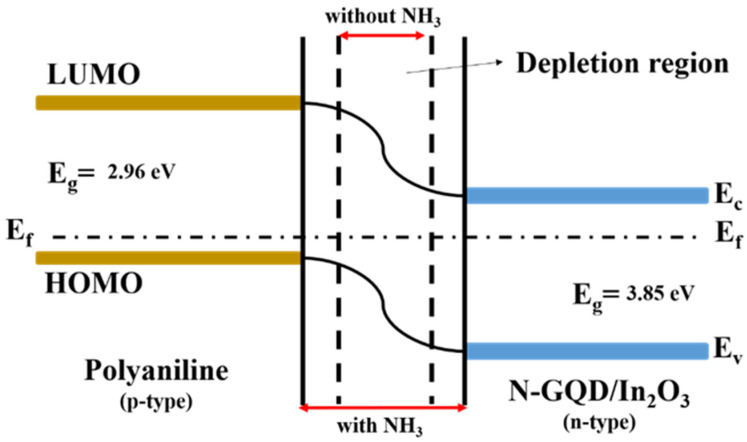
The schematic illustration of the sensing mechanism and energy band gap structure diagram of PANI/GQD/hollow In_2_O_3_ nanofiber composite sensors.

The PANI/N-GQD/hollow In_2_O_3_ nanofiber sensor was applied to detect NH_3_ in a concentration ranging from 0.6 ppm to 2.0 ppm at room temperature and to estimate the NH_3_-sensing performance of the composite sensor used in the analysis of human breath for hepatic or kidney disease. The NH_3_-sensing performance of the PANI/N-GQD/hollow In_2_O_3_ nanofiber sensor compared with the PANI and PANI/hollow In_2_O_3_ nanofiber sensors is shown in Figure 9a. These results indicate that the response of each sensor immediately increased as the exposure to NH_3_ increased and then dropped significantly to an initial state after exposure to dry air. When the concentration of the analyte increased, the response of each sensor increased significantly. These results suggest that all responses of the three sensors had almost the same trend but the response values of the three sensors at the same concentration were exceptionally different. The response values of the PANI/N-GQD/hollow In_2_O_3_ nanofiber sensors were about 9.4, 11.6, 15.2, 17.4, 22.7, 28.6, 35.8, and 41.2 toward to the corresponding concentrations of 0.6, 0.8, 1.0, 1.2, 1.4, 1.6, 1.8, and 2.0 ppm, respectively. It is apparent that the PANI/N-GQD/hollow In_2_O_3_ nanofiber sensor exhibited the highest response among the three sensors. At 1.0 ppm, the response value of the PANI/N-GQD/hollow In_2_O_3_ nanofiber sensor was about 4.4 and 1.4 times higher than those of the PANI and PANI/hollow In_2_O_3_ nanofiber sensors, respectively. Figure 10 shows the responses of the three sensors as a function of various concentrations of NH_3_. These curves reveal that the correlation between the response values and the NH_3_ concentration is nearly linear. The corresponding functions were determined as y = 2.04x + 2.55, y = 15.23x − 3.88, and y = 23.95x − 10.41 for the PANI, PANI/hollow In_2_O_3_ nanofiber, and PANI/N-GQD/hollow In_2_O_3_ nanofiber sensors, respectively. The correlation coefficients, R^2^, were approximately equal to 0.994. The slopes of the corresponding lines, designated as the sensitivities of the sensors, reveal that the sensitivity of the PANI/N-GQD/hollow In_2_O_3_ nanofiber sensor was superior to those of the PANI and PANI/hollow In_2_O_3_ nanofiber sensors. These findings suggest that the PANI/N-GQD/hollow In_2_O_3_ nanofiber sensor has a greater ability to detect NH_3_, recommending it for suitable use as a favored substance for NH_3_ gas detection. A comparison of the sensing performance of the PANI/N-GQD/hollow In_2_O_3_ nanofiber sensor and previously described devices was recorded in Table 2. It is evident that the PANI/N-GQD/hollow In_2_O_3_ nanofiber sensor exhibited better sensing performance to NH_3_ at room temperature than previously described devices. Consequently, commingled p-type PANI and n-type N-GQD-coated hollow In_2_O_3_ nanofibers could be applied as an effectual approach for improving the NH_3_-sensing response of sensors.

Figure 10 presents the synergistic effect of the PANI/N-GQD/hollow In_2_O_3_ nanofiber ternary material, from which it is apparent that the combination of N-GQD and hollow In_2_O_3_ nanofibers enhances the response of PANI to NH_3_ gas. PANI-coated N-GQD/hollow In_2_O_3_ nanofibers can generate new chemical bonds on the oxygen-containing defects of N-GQD and hollow In_2_O_3_ nanofiber surfaces. The extensive special surface area of N-GQDs and hollow In_2_O_3_ nanofibers further improves the contact sites with PANI, which can provide a considerable number of adsorption sites for NH_3_ gas. This observable fact has previously been proved in Figure 4, Figure 5, and Figure 7. Consequently, the synergistic arrangement of N-GQD, hollow In_2_O_3_ nanofiber, and PANI creates exceptional sensing properties superior to those of pure PANI and PANI/hollow In_2_O_3_ nanofiber sensors. The characteristic of p–n heterojunctions generated between the p-type PANI and n-type N-GQD-coated hollow In_2_O_3_ nanofibers could be attributed as another NH_3_-sensing mechanism of the PANI/N-GQD/hollow In_2_O_3_ nanofiber film. The positions of the conduction band (E_c_) and valence band (E_v_) in In_2_O_3_, the highest occupied molecular orbital (HOMO) and lowest unoccupied molecular orbital (LUMO) for PANI, and the Fermi levels (E_f_) regarding the vacuum level are presented in the energy band gap structure diagram in Figure 10. The band gaps for PANI and In_2_O_3_ are 2.96 and 3.85 eV, respectively [15,37]. As the occurrence of the heterojunction between the p-type PANI and n-type N-GQD-coated hollow In_2_O_3_ nanofiber is present, a self-established electronic field of depletion layer is generated. When the sensor is subjected to NH_3_ gas, the In_2_O_3_ electrons and PANI holes shift in opposite directions until the new Fermi level achieves equilibrium. During this procedure, electron transfer from the n-type N-GQD-coated hollow In_2_O_3_ nanofiber to the p-type PANI is delayed, attributable to the potential barrier, which enlarges the depletion layer thickness and resistance of the sensor [37]. The p–n heterojunction performs as an indicator amplifier and makes it easier to effectively detect tiny quantities of NH_3_ [38].

**Figure 11:**
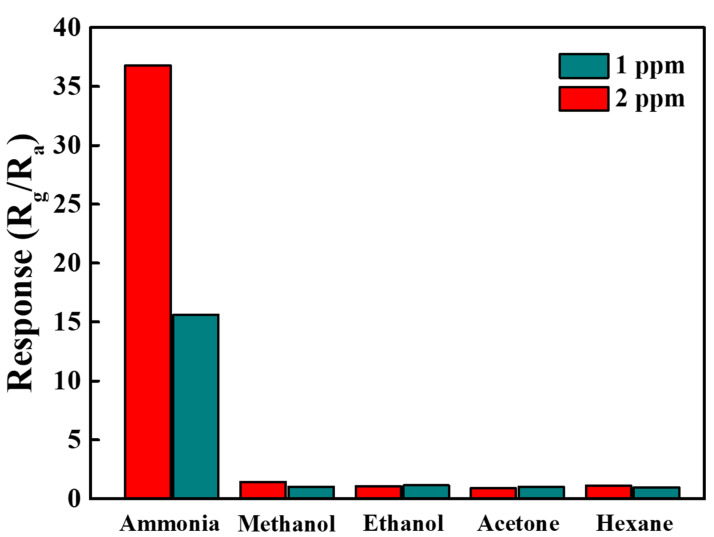
The selectivity of the PANI/GQD/hollow In_2_O_3_ nanofiber film sensor tested at the concentrations of 1 and 2 ppm toward NH_3_, methanol, ethanol, and acetone.

For practical applications, the repeatability, reversibility, and selectivity of synthesized gas sensors are critical. Actually, gas sensors are normally exposed to numerous analytes, and it is assumed that the target analyte is detected accurately without being influenced by other species. Figure 11 exhibits the selectivity of the PANI/N-GQD/hollow In_2_O_3_ nanofiber sensor toward ammonia (NH_3_), methanol (CH_3_OH), ethanol (C_2_H_5_OH), and acetone (CH_3_COCH_3_) with a concentration of 1.0 and 2.0 ppm. The selectivity test shown in Figure 11 is the first test in sensor characterization. It was noticeably illustrated that the PANI/N-GQD/hollow In_2_O_3_ nanofiber sensor had a high-level response property to ammonia but displayed approximately no response to other gases. The mechanism of NH_3_ selectivity may be related to the surface absorption of NH_3_ on the interface of the PANI/N-GQD/hollow In_2_O_3_ nanofiber sensor. The de-doping response between NH_3_ and PANI plays an important role in improving NH_3_-sensing performance, leading to a selective response to NH_3_ [18,34]. Subsequently, it can be established that the PANI/N-GQD/hollow In_2_O_3_ nanofiber sensor exhibited selectivity toward NH_3_ at room temperature as against other gases.

## 4. Conclusions

We have shown outstanding gas-sensing performances of PANI/N-GQD/hollow In_2_O_3_ nanofiber ternary composites effectively synthesized by in situ chemical oxidation polymerization. The response value of the 20 wt% N-GQD-coated hollow In_2_O_3_ nanofiber added to the PANI/N-GQD/hollow In_2_O_3_ nanofiber sensor was 15.1 at an exposure of 1 ppm NH_3_, which was approximately 4.4 and 1.4 times higher than those of the PANI and PANI/hollow In_2_O_3_ nanofiber sensors, respectively. The PANI/N-GQD/hollow In_2_O_3_ nanofiber sensor showed the highest response at room temperature in detecting NH_3_ concentrations ranging from 0.6 ppm to 2.0 ppm, as compared to those of the PANI and PANI/hollow In_2_O_3_ nanofiber sensors; this is critical for detecting hepatic or kidney disease in human breath. The PANI/N-GQD/hollow In_2_O_3_ nanofiber sensor also showed higher repeatability and selectivity on exposure to 1.0 and 2.0 ppm NH_3_ at room temperature. Owing to the exceptional repeatability and selectivity in the detection of 1.0 and 2.0 ppm NH_3_ at room temperature reported in this study, it is considered that the PANI/N-GQD/hollow In_2_O_3_ nanofiber composite sensor will be a favored gas-sensing material for the detection of hepatic or kidney disease in human breath.

## Figures and Tables

**Table 1:** Data of the specific surface area and the coating thickness of the PANI layer for the PANI/hollow In_2_O_3_ nanofiber composites.

Sample	Specific Surface Area (m^2^/g)	Coating Thickness of PANI (nm)
2.5 wt% PANI/hollow In_2_O_3_ nanofiber	25.0	56.1
5 wt% PANI/hollow In_2_O_3_ nanofiber	47.7	23.7
10 wt% PANI/hollow In_2_O_3_ nanofiber	48.3	19.4
15 wt% PANI/hollow In_2_O_3_ nanofiber	69.9	17.7
20 wt% PANI/hollow In_2_O_3_ nanofiber	102.1	10.3
25 wt% PANI/hollow In_2_O_3_ nanofiber	73.5	19.6

NH_3_-sensing performance of PANI/N-GQD/hollow In_2_O_3_ nanofiber composites.

**Table 2:** Comparison of NH_3_-sensing performance of the PANI/N-GQD/hollow In_2_O_3_ nanofiber sensor developed here with other sensors reported recently.

Materials	Gas	Conc. (ppm)	Temp. (°C)	Response (%)	Ref.
PANI/WO_3_	NH_3_	10	RT	7.14	[14]
PANI/In_2_O_3_	NH_3_	100	RT	3.2	[35]
PANI/TiO_2_-SiO_2_	NH_3_	50	RT	10	[39]
PANI/CeFe_2_O_4_	NH_3_	50	RT	118.3	[17]
PANI/Graphene	NH_3_	50	RT	14.6	[40]
PANI/GNR/In_2_O_3_	NH_3_	0.65	RT	8.6	[18]
PANI/PMMA	NH_3_	1	RT	1.4	[41]
PANI/Graphene/SnO_2_	NH_3_	10	RT	2.8	[42]
PANI/MoS_2_/SnO_2_	NH_3_	50	RT	7.5	[43]
PANI/N-GQD/hollow In_2_O_3_ nanofiber	NH_3_	1	RT	15.6	This work

## Data Availability

Not applicable.
